# Risk of tuberculosis is increased in Behçet’s disease compared to other rheumatological disorders after anti-TNFα treatments: a case series and review of the literature

**DOI:** 10.3906/sag-2010-311

**Published:** 2021-08-30

**Authors:** Ümmügülsüm GAZEL, Derya KOCAKAYA, İrem HİCRET TOPÇU, Hakan ÖMER KARATAŞ, Murat KARABACAK, Mehmet Pamir ATAGÜNDÜZ, Güzide Nevsun İNANÇ, Fatma ALİBAZ ÖNER, Rafi Haner DİRESKENELİ

**Affiliations:** 1 Department of Rheumatology, Faculty of Medicine, Marmara University, İstanbul Turkey; 2 Department of Pulmonary and Critical Care Medicine, Faculty of Medicine, Marmara University, İstanbul Turkey; 3 School of Medicine, Marmara University, İstanbul Turkey

**Keywords:** Behçet’s disease, TNF-a antagonists, tuberculosis

## Abstract

**Background/aim:**

Tumor necrosis factor-alfa (TNF-a) antagonists are extensively utilized in the treatment of inflammatory rheumatic diseases and also shown to be effective in Behçet’s disease (BD) patients with major organ involvement. In this study, we aimed to re-evaluate the incidence of tuberculosis (TB) infection after anti-TNFa treatments and to reveal the risk of TB in BD.

**Methods:**

Data of patients who received anti-TNFa treatment between 2005 and 2018 were assessed retrospectively. Demographic features, TNF-a antagonist type/treatment time, tuberculosis skin test (TST) and QuantiFERON results, isoniazid prophylaxis status, and concomitant corticosteroid (CS) treatments were collected.

**Results:**

A total of 1277 (male/female = 597/680; median age = 49 years) patients were treated with TNF-a antagonist for a median of 33 months (Q1:12, Q3:62). Thirteen (1%) patients developed TB during the follow-up period. Within 13 TB-positive patients, 7 of them had pulmonary, and 7 had extrapulmonary TB. Although, the median time of (month) TNF-a antagonist treatment was higher in TB-positive patients than negative ones, the difference was not statistically significant (48 and 33 months, respectively, p = 0.47). Similarly, TB-positive patients were treated with CSs more than TB-negative patients (80% vs. 60%). Time from the initiation of TNF-a antagonist treatment to the diagnosis of TB had a median of 40 months (Q1-Q3: 22-56). There was a statistically significant increase of TB development in BD patients than non-BD patients after TNF-a antagonists (7.5% vs. 0.8%, respectively, p = 0.007). When we combined our patients with the other series from Turkey, among 12928 patients who received TNF-a antagonists, TB was positive in 12 (3.9%) of 305 BD patients compared to 112 (0.9%) of 12623 non-BD patients (p < 0.00001).

**Conclusion:**

Our results suggest a higher frequency of TB infections in BD patients with TNF-a antagonists. As biologic agents are increasingly used for major organ involvement in current practice for BD, screening mechanisms should be carefully implemented.

## 1. Introduction 

Tumor necrosis factor-alfa (TNF-a) antagonists are extensively utilized in the treatment of inflammatory rheumatic diseases such as rheumatoid arthritis (RA) and ankylosing spondylitis (AS). In recent years, experience with these agents has also increased in other immune rheumatological diseases. In Behçet’s disease (BD), a multisystemic inflammatory disorder, TNF-a antagonists are also shown to be effective in patients with major organ involvement, refractory to conventional immunosuppressives (IS) [1].

The risk of infections is shown to be increased with the use of TNF-a antagonists as a significant side effect [2]. The enhanced risk of mycobacterial infections is especially important in countries with still an elevated prevalence of tuberculosis (TB). In a multicenter large cohort from Turkey, it was previously observed that patients with BD are more likely to develop TB after anti-TNFa treatments compared to other rheumatic diseases [3]. The underlying mechanism of this observation may be related to factors unique to BD, such as genetics, immune mechanisms, or socio-economic status, as well as concomitant treatment with high dose corticosteroid (CS) and immunosuppressive therapies [4,5].

In this study, we aimed to re-evaluate the incidence of TB infection after anti-TNFa treatments for rheumatic diseases and to reveal the risk of TB according to the rheumatologic diagnosis, particularly BD. Factors associated with this risk are also assessed.

## 2. Methods 

### 2.1. Patients and data collection 

The data of 1277 patients who received TNF-a antagonists between 2005 and 2018 at Marmara University, School of Medicine, Rheumatology Outpatient Clinics, were assessed retrospectively. Patients had to have an inflammatory rheumatic disease treated with one of the TNF-a antagonists (Adalimumab, Infliximab, Etanercept, Golimumab, and Certolizumab Pegol) to be included in the study. Follow-up data were collected from standard patient report forms and medical chart reviews: demographic features, TNF-a antagonist type and treatment time, tuberculosis skin test (TST) (positive/negative) or QuantiFERON results, isoniazid prophylaxis status, concomitant CS treatment (yes/no). For TB patients, time until TB development and organ involvement type were also recorded.

### 2.2. Tuberculosis (TB) screening 

Patients were screened for TB before and during the TNF-a antagonist treatments according to the Consensus Report on using TNF-a antagonists endorsed by the Rheumatology Society of Turkey [6]. Before starting TNF-a antagonists, patients were evaluated for TB by a questionnaire for the presence of prior TB infection or treatment with antituberculosis drugs and a history of contact with a tuberculosis-infected case. Additionally, patients were evaluated for the occurrence of TB signs and symptoms with a physical examination. At the same time, patients underwent TST or QuantiFERON test depending on the availability and Chest radiograph. If the TST result is ≥ 5mm or QuantiFERON test was positive, isoniazid (INH) prophylaxis 300 mg/day for nine months was started. In case of a suspicious finding for latent TB in the chest radiograph or in physical examination and anamnesis, INH prophylaxis was decided with a chest disease or infectious disease specialist, even if screening tests were negative. Patients were followed up every 3–6 months for the development of pulmonary or extra-pulmonary TB with symptom inquiry and physical examination. Chest radiographs were also repeated once a year. In the case of suspected TB infection during follow-up, an appropriate evaluation was made. 

### 2.3. Statistics

Means of continuous variables and standard deviation when the data were distributed normally were calculated. Medians and first to third quartile intervals were calculated when data that did not have a normal distribution. Group differences for dependent categorical variables were analyzed with the Chi-square or Fishers’ exact test. Continuous variables were compared with student t-test or Mann–Whitney U when data were skewed. A two-tailed p-value < 0.05 was considered significant.

## 3. Results

### 3.1. Patient demographics 

A total of 1277 (male/female = 597/680; median age = 49 years) patients were treated with TNF-a antagonist for a median of 33 months (Q1:12, Q3:62). During this period, 1560 different TNF-a antagonist prescriptions were started, Etanercept (n = 509, 33%) and Adalimumab (n = 509, 33 %) were the most frequently selected. The most common diagnosis were AS (n = 644; 51%) and RA (n = 477; 38%), respectively. Either TST or QuantiFERON tests were positive in 823 (65%) patients, and the rate of patients that used INH prophylaxis (872, 69%) was slightly more than test positivity. CS treatment data were collected in 795 patients as received/not received. Within this group, 38% of the patients received CS at any time during the follow-up period. The characteristics of all patients in the study are summarized in Table 1. 

**Table 1:** Characteristics of the patients treated with anti-TNF-α antagonists.

	All patients(n = 1277)	Patients without TB(n = 1264)	Patients with TB(n = 13)	p*
Age median (Q1-Q3), years	49 (39–58)	49 (39–58)	50 (41–61)	0.514
Male	597 (47)	587 (46.4)	10 (77)	0.046
TST and/or QuantiFERON Positivity	823 (65)	815 (65)	8 (61)	0.530
INH prophylaxis	875/1268 (69)	867/1255 (69)	8/13 (61)	0.556
Corticosteroid treatment (at any time)	480/797 (60)	472/785 (60)	8/10 (80)	0.330
Anti-TNFa treatment time median (Q1-Q3) (month)	33 (12–62)	33 (12–62)	48(22–80)	0.470
Anti-TNFa treatment­				
Etanercept	509 (40)	501 (40)	8 (61)	0.153
Adalimumab	509 (40)	504 (40)	5 (38)	1.000
Infliximab	380 (30)	375 (30)	5 (38)	0.544
Certolizumab	84 (7)	81 (6)	3 (23)	0.049
Golimumab	78 (6)	78 (6)	0	1.000
Inflammatory disease				
Rheumatoid arthritis	479 (38)	475 (38)	4 (30)	0.777
Ankylosing spondylitis	648 (51)	643 (51)	5 (38)	0.415
Psoriatic arthritis	96 (8)	95 (8)	1 (8)	1.000
Behçet’s disease	40 (3)	37 (3)	3 (23)	0.007
Takayasu’s arteritis	14 (1)	14 (1)	0	1.000

Except where indicated otherwise, values are the number/total number (percent) of patients. TNF: tumor necrosis factor; TB: tuberculosis; TST: tuberculosis skin test; INH: isoniazid.

### 3.2. Patient characteristics with and without tuberculosis (TB)

Among 1277 patients, 13 (1%) of them developed TB during the follow-up period. While the median age of TB-positive and negative patients was similar (50 and 49 years, respectively), male presence was higher in TB-positive patients (76% vs. 24%). TST and/or QuantiFERON positivity and INH usage rates were comparable between the two groups. Although, the median time of (month) TNF-a antagonist treatment was higher in TB-positive patients than negative ones, the difference was not statistically significant (48 and 33 months, respectively, p = 0.47). Similarly, TB-positive patients were treated with CS more than TB-negatives (80 % vs. 60 %). The comparison of patients with and without TB is summarized in Table 1. All percentages are given based on the total number of patients with and without TB within the columns in Table 1.

Within 13 TB-positive patients, 6 of them had pulmonary, 6 had extra-pulmonary TB and 1 had both. Time from the initiation of TNF-a antagonist treatment to the diagnosis of TB had a median of 40 months (Q1-Q3: 22-56). At the time of TB diagnosis, all 13 patients were on anti-TNFa treatments (4 Etanercept, 5 Adalimumab, 2 Infliximab, and 2 Certolizumab Pegol). Due to disease activity, Certolizumab Pegol and Etanercept were started again after TB treatment for two patients with AS diagnosis.

According to the underlying disease, TB was more frequent in the AS group (n = 5, 0.7%) compared to the other diseases, but patients with BD (n = 3, 8.1%) had the highest rate for TB (Table 2). There was a statistically significant increase of TB development in BD patients than non-BD patients after TNF-a antagonists (7.5% vs. 0.8%, respectively, p = 0.007). When we combined our patients with the other series from Turkey, among 12928 patients who received TNF-a antagonists, TB was positive in 12 (3.9%) of 305 BD patients compared to 112 (0.9%) of 12623 non-BD patients (p < 0.00001). A summary of all studies published from Turkey, which reported the TB incidence after TNF-a antagonists and comparison with the present study is given in Table 3.

**Table 2:** The frequency of TB development according to the underlying disease.

Disease	Patients with TB(n)	Patients without TB(n)	%
Rheumatoid arthritis	4	475	0.8
Ankylosing spondylitis	5	643	0.7
Psoriatic arthritis	1	95	0.1
Behçet’s disease	3	37	8.1
Takayasu’s arteritis	0	14	-

**Table 3:** Studies comparing tuberculosis risk in Behçet’s disease to other inflammatory diseases in the published literature.

	Börekçi et al., 2015	Kisacik et al., 2016	Çağatay et al.,2017	Current study
Total number of patients treated with TNF-α antagonists (n)	1964	7768	1887	1277
Age years (mean ± SD)	39.7 ± 13.9	43.4 ± 13.6	42.41 ± 12.71	48.7 ± 13.2
Male/Female(n)	1009/955	3673/4095	907/980	597/680
TST/ QuantiFERON positivity, n (%)	1162 (59)	4243 (54.6)	1127 (67.4)	823 (65)
INH prophylaxis n (%)	1250 (63.6)	5704 (73.4)	NR	872 (69)
Time from initiation of TNF-a antagonist treatment to the diagnosis of TB	mean ± SD:26.8 ± 17.1 months(range 1–60, median 26.5)	IFX: 13 months(range 1–96),ADA: 13 months (range 3–36),ETN: 7 months (range 4–60)	mean ± SD:25.7 ± 19.4	median: 40 months(Q1-Q3: 22–56)
TB positivity, n (%)Behçet’s disease Other diseases	3/83 (3.6)13/1881 (0.7)	5/129 (4)68/7639 (0.8)	1/53 (1.8)21/1866 (1.1)	3/40 (7.5)10/1237 (0.8)

TNF: tumor necrosis factor; TB: tuberculosis; TST: tuberculosis skin test; INH: isoniazid; NR: not reported; IFX: infliximab; ADA: adalimumab; ETN: etanercept; SD: standard deviation.

### 3.3. Comparison of BD patients with and without tuberculosis 

Among 40 patients with BD, TB-positive patients (n = 3) were all male and had a slightly younger mean age than TB-negatives (34.6 vs. 39 years, respectively, p = 0.456). All TB-positive patients with BD used INH prophylaxis and had a positive TST/QuantiFERON. Median time (month) of TNF-a antagonist and CS treatment rates were also slightly higher in TB-positive than TB negative patients with BD [(20 months vs 16 months, respectively, p = 0.939) and (3/3 100% vs 29/33 88%, respectively, p = 0.522). 

## 4. Discussion

This study confirmed the increased risk of TB after anti-TNFa treatments and suggested that this increased risk might be greater in BD compared to other rheumatic disorders. 

TNF-a has an important role, particularly in the defense against mycobacteria and is essential for the maintenance of granuloma formation [7]. Other key functions of TNF-a include regulation of apoptosis, release of cytokines from activating macrophages, and effects on the formation of nitric oxide and the leucocyte movement [8]. Increased TB frequency after anti-TNFa treatments has been reported from large registries [9–12]. This increased risk has been reported more prominently for monoclonal antibodies Adalimumab and Infliximab compared to soluble receptors such as Etanercept. Although screening and prophylaxis improved much in recent years, TNF-a inhibition may affect some countries more seriously, such as Turkey, with a not too-low (22/100 000) incidence of TB. In a multicenter cohort study from Turkey, Kisacik et al. reported 73 TB-positive cases within 7768 patients treated with TNF-a antagonists [3]. In other publications from Turkey, Çağatay et al*.* reported 22 TB-positive cases within 1887 patients and Börekçi et al. 16 TB-positive cases within 1964 patients after TNF-a antagonists [13,14]. Our TB prevalence was similar to these other studies from Turkey (0.7-1.1%).

As both BD and TB infection are common diseases in Turkey, TB data among TNF-a antagonist treated patients deserve separate analysis. Apart from Turkey, a few case reports have been published in the literature on BD and TB coexistence [15-18]. In the series from Turkey, Kisacik et al. showed that TB development rate was higher in BD compared to other rheumatic diseases (5/129, 4%). TB rate was also reported higher in BD than other diseases by Çağatay et al. (1/53, 1.8%). Börekçi et al. also found BD to be associated with the development of TB infection in multivariate analysis (3/83, 3.6%, p = 0.003). We documented even a higher rate of TB among BD patients than other Turkish cohorts (3/40, 7.5 %). As a whole, the more frequent use of TNF-a antagonists in recent years in patients with major organ involvement, also suggested by the recent EULAR recommendations, requires more research on the infectious side-effects of anti-TNFa treatments in BD (1). 

Some underlying mechanisms may be suggested for this higher TB prevalence after anti-TNFa exposure in BD. Patients with major-organ BD are generally males at a younger age and usually actively participate in the workforce, which may cause more contact with mycobacteria compared to other patient groups. The fact that BD patients have lower socio-economic status may be another reason [4,5]. Higher CS doses (0.5-1 mg/kg/day), especially used in the initial use of TNF-a antagonists, to suppress major organ damage such as vision loss or arterial complications may also contribute to the development of TB. Immune mechanisms leading to BD activation, such as augmented innate immunity and male sex-related effects, might also predispose BD patients to mycobacterial infections. Finally, some HLA genes are associated with tuberculosis [19–22]. Some of these genetic associations are also shown in BD [23,24]. More studies are needed to clarify these mechanisms.

Our study has some limitations. Data for the concomitant use, especially dosing of CS treatments and additional immunosuppressives, are not collected in our group. Also, we do not formally assess the socio-economic status data of our patients. 

In conclusion, our results suggest a higher frequency of TB infections in BD patients with TNF-a antagonists. As biologic agents are increasingly used for major organ involvement in current practice for BD, screening mechanisms should be carefully implemented and followed. 

## Informed consent 

Study protocol was approved by Marmara University Local Ethics Committee (approval number: 09.2020.978) and the study was performed according to the Declaration of Helsinki. Patient informed consent was not required for being a retrospective data collection.
